# CroMaSt: a workflow for assessing protein domain classification by cross-mapping of structural instances between domain databases and structural alignment

**DOI:** 10.1093/bioadv/vbad081

**Published:** 2023-06-27

**Authors:** Hrishikesh Dhondge, Isaure Chauvot de Beauchêne, Marie-Dominique Devignes

**Affiliations:** Université de Lorraine, CNRS, Inria, LORIA, F-54000 Nancy, France; Université de Lorraine, CNRS, Inria, LORIA, F-54000 Nancy, France; Université de Lorraine, CNRS, Inria, LORIA, F-54000 Nancy, France

## Abstract

**Motivation:**

Protein domains can be viewed as building blocks, essential for understanding structure–function relationships in proteins. However, each domain database classifies protein domains using its own methodology. Thus, in many cases, domain models and boundaries differ from one domain database to the other, raising the question of domain definition and enumeration of true domain instances.

**Results:**

We propose an automated iterative workflow to assess protein domain classification by cross-mapping domain structural instances between domain databases and by evaluating structural alignments. CroMaSt (for Cross-Mapper of domain Structural instances) will classify all experimental structural instances of a given domain type into four different categories (‘Core’, ‘True’, ‘Domain-like’ and ‘Failed’). CroMast is developed in Common Workflow Language and takes advantage of two well-known domain databases with wide coverage: Pfam and CATH. It uses the Kpax structural alignment tool with expert-adjusted parameters. CroMaSt was tested with the RNA Recognition Motif domain type and identifies 962 ‘True’ and 541 ‘Domain-like’ structural instances for this domain type. This method solves a crucial issue in domain-centric research and can generate essential information that could be used for synthetic biology and machine-learning approaches of protein domain engineering.

**Availability and implementation:**

The workflow and the Results archive for the CroMaSt runs presented in this article are available from WorkflowHub (doi: 10.48546/workflowhub.workflow.390.2).

**Supplementary information:**

Supplementary data are available at *Bioinformatics Advances* online.

## 1 Introduction

**Figure 1.**
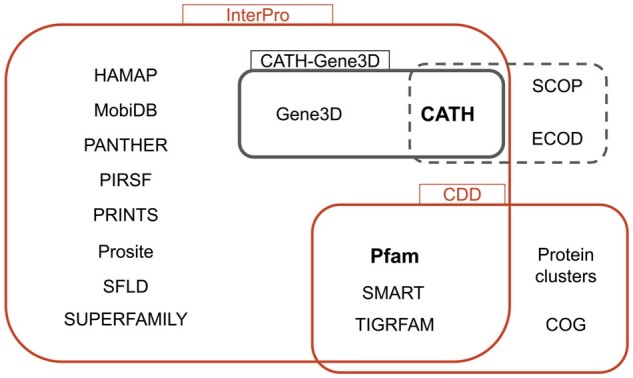
General landscape of domain databases. In red are the integrated domain databases (InterPro and CDD). CATH, SCOP and ECOD are structure-based domain databases (enclosed in dashed line) and the rest are sequence-based domain databases

Most proteins are composed of one or more domains that can be identified at the sequence or structural level. A protein domain is defined by Bateman *et al.* (2002) as ‘an autonomous structural unit, or a reusable sequence unit, that may be found in multiple protein contexts’. It generally corresponds to a structural unit of a protein that can fold independently of the rest of the protein (Kelley and Sternberg, 2015). Most often, a domain type can be associated with a specific, definite function in the proteins, making it the basic unit for understanding protein function and building synthetic proteins with given functions. Domains can be recognized in a protein either by *ab initio* methods capable of detecting domain boundaries, or by classification methods based on sequence or structure characteristics shared between the different members of a given domain family. There are several resources available that provide information about the protein domains and their classification (Wang *et al.*, 2021). The domain databases can be grouped into three categories depending on the rationale used for classification, which can be either primarily sequence-based, primarily structure-based or integration-based (Supplementary Section A). Figure 1 summarizes the general landscape of domain databases. In red are the integrated domain databases: InterPro (Paysan-Lafosse *et al.*, 2023) and CDD (Lu *et al.*, 2020). CATH (Sillitoe *et al.*, 2021), SCOP (Andreeva *et al.*, 2020) and ECOD (Cheng *et al.*, 2014) are structure-based domain databases (framed with a dashed outline) and the rest are sequence-based domain databases. Pfam (Mistry *et al.*, 2021) and CATH (in bold font) are used in this study.

All these domain databases constitute valuable and complementary sources of knowledge about domain families and can be used to investigate structure–function relationships in proteins. However, several problems arise when one begins to examine a particular domain type across multiple domain databases. Indeed, due to the domain diversity and the variety of classification rationales, a given domain type can be represented by one to several domain families in databases. The first problem encountered is that these domain families have different names in different databases and are not consistently mapped to each other. Secondly, domain families corresponding to the same domain type in two different databases may not contain the same domain instances. Thirdly, some family members can be wrongly assigned to the given domain type. Finally, for a given family member present in two different domain databases, the domain boundaries on the sequence (start and end residue positions) may be different.

Such difficulties are particularly deleterious for domain-centric investigation in the frame of synthetic biology and protein design. Indeed, such projects require a very precise knowledge about existing instances of a given domain type associated with a specific function, in order to be able to engineer synthetic versions of this domain type without losing the function. In practice, the exhaustive enumeration of all true domain instances of a certain domain type is a complex problem and cannot be solved by querying a single domain database. To solve these issues, we propose here a generic iterative approach aiming to clarify the assignment of domains to a given domain type using their membership to domain families from various databases and the evaluation of their structural alignment with a typical representative 3D structure.

**Figure 2.**
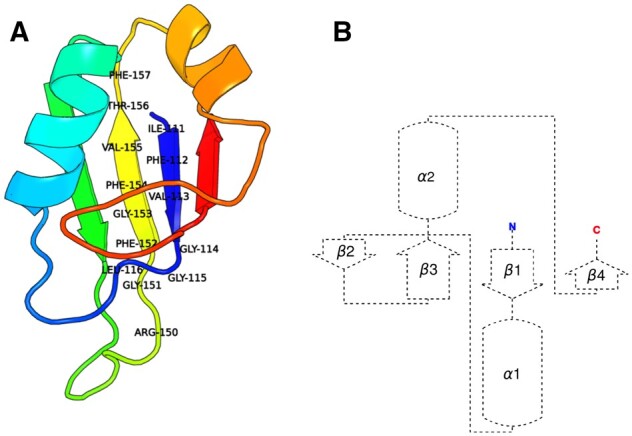
Typical characteristics of RRM domain illustrated with PDB entry 2MSS (Musashi1 RBD2, NMR). The amino acids of conserved motifs RNP1 (RGFGFVTF) and RNP2 (IFVGGL) present in β3 and β1 segments, respectively, have been indicated. (**A**) Structure of the ‘2MSS’ RRM domain with conserved RNP sequences and (**B**) topology of the ‘2MSS’ RRM domain

As a use-case to develop and test our approach, we use the RNA Recognition Motif (RRM) domain type, with the goal to integrate all existing information about it, including all available experimentally determined 3D structures. RRM domains are ∼90 amino acids long, with a typical β1α1β2β3α2β4 topology that forms a four-stranded β-sheet packed against two α-helices, and with two consensus sequences known as RNP1 and RNP2. The RNP1 and RNP2 consensus sequences are (R/K)-G-(F/Y)-(G/A)-(F/Y)-V-X-(F/Y) and (L/I)-(F/Y)-(V/I)-X-(N/G)-L, located on β3 and β1, respectively (Maris *et al.*, 2005) (Fig. 2). All these features are characteristics of the RRM domain type. Proteins containing RRM domains can bind to specific RNA sequences. Consequently, RRM domains play an important role in several key biological processes including post-transcriptional gene regulation, abnormal cell proliferation (Chen *et al.*, 2019), maintenance of stem cells and telomerase activity (Xie *et al.*, 2021). Thus, RRM domain engineering has many applications for the creation of new synthetic biological pathways and for the discovery of new treatments for RNA-associated diseases (Shotwell *et al.*, 2020).

Inconsistencies regarding RRM domain families appear with a very simple search using keyword ‘RRM’. There are 16 Pfam families and two CATH superfamilies with RRM in their name (for a search performed on Pfam version 35.0 and CATH version 4.3.0). Only 13/16 Pfam families are listed in the Pfam RRM clan. In CATH, several domains matching keyword ‘RRM’ were retrieved from outside the two ‘RRM’ superfamilies (see query details in Supplementary Section B).

Thus, different classification systems provide very different results for a given domain type, such as RRM. Although the RRM domain type appeared well characterized in early studies, a great diversity exists today among domain families referring to RRMs. This raises the question of the allowed diversity among domains assigned to a given domain type: which domain is a ‘true’ RRM domain type and which one is not. In this article, we decided to answer to this question from a 3D structure point of view and we designed a generic systematic approach: CroMaSt, that classifies all structures possibly corresponding to a given domain type into four different categories (‘Core’, ‘True’, ‘Domain-like’ and ‘Failed’). These structures are hereafter named structural instances (StIs) and a given protein domain sequence may have more than one StIs. We first describe our approach including our cross-mapping concept. Then, Section 3 details the implementation of the CroMaSt generic workflow and is followed by Section 4 with RRM domain type as a use-case. Finally, we will discuss the possible usage of the CroMaSt workflow.

## 2 Approach

Our objective is to clarify the assignment of protein domains to a given domain type of interest, based on their 3D structure. The CroMaSt workflow is currently designed to be used with the domain classifications from two well-known and widely used domain databases, Pfam (primarily sequence-based) and CATH (primarily structure-based). Our generic approach assumes that there exists a consensus basic definition of the considered domain type, with a few known 3D structures qualified by experts as representative StIs (see Section 3.2) for this domain type. Then, our first hypothesis is that it is possible to verify that a given domain StI belongs to the considered domain type by running structural alignment with these representative StIs. The result can be either manually inspected or automatically filtered using appropriate thresholds defined by the experts of this domain type. However, this task can become tedious in view of the ever-increasing number of StIs. Therefore, one needs to rely on existing domain classifications, in particular those with wide coverage, such as CATH and Pfam. Let CATH-rep1 and Pfam-rep1 be two most representative domain families in CATH and Pfam databases, respectively. Our second hypothesis is that if a domain StI belongs to both CATH-rep1 and Pfam-rep1, then it is likely to be a ‘True’ StI. If a domain StI is only present in CATH-rep1 (respectively in Pfam-rep1), it can be relevant to ‘cross-map’ (process to map a given StI from one database to a family in a target database; see Section 3.4) it to another domain family in Pfam database (respectively in CATH database), and to check all the StIs of this new domain family that may become a new representative family of the considered domain type.

At the end of the process, four categories of domain StIs are produced (Table 1): the ‘Core’ category groups all domain StIs that are present in both initial representative domain families (one from each database), the ‘True’ category groups all domain StIs that have been successfully cross-mapped and show significant structural similarity to the 3D representatives of the domain type, the ‘Domain-like’ category groups all domain StIs that could not be cross-mapped but show a significant structural similarity and the ‘Failed’ category groups all domain StIs that do not show a significant structural similarity (either cross-mapped or un-mapped).

**Table 1.** Rationale behind assigning categories to domain StIs with respect to a given domain type

Category	Shared between starting families	Cross-mapped	Significant structural similarity
Core	✓	—	—
True	✗	✓	✓
Domain-like	✗	✗	✓
Failed	✗	✓ or ✗	✗

*Note*: —Not applicable.

**Figure 3.**
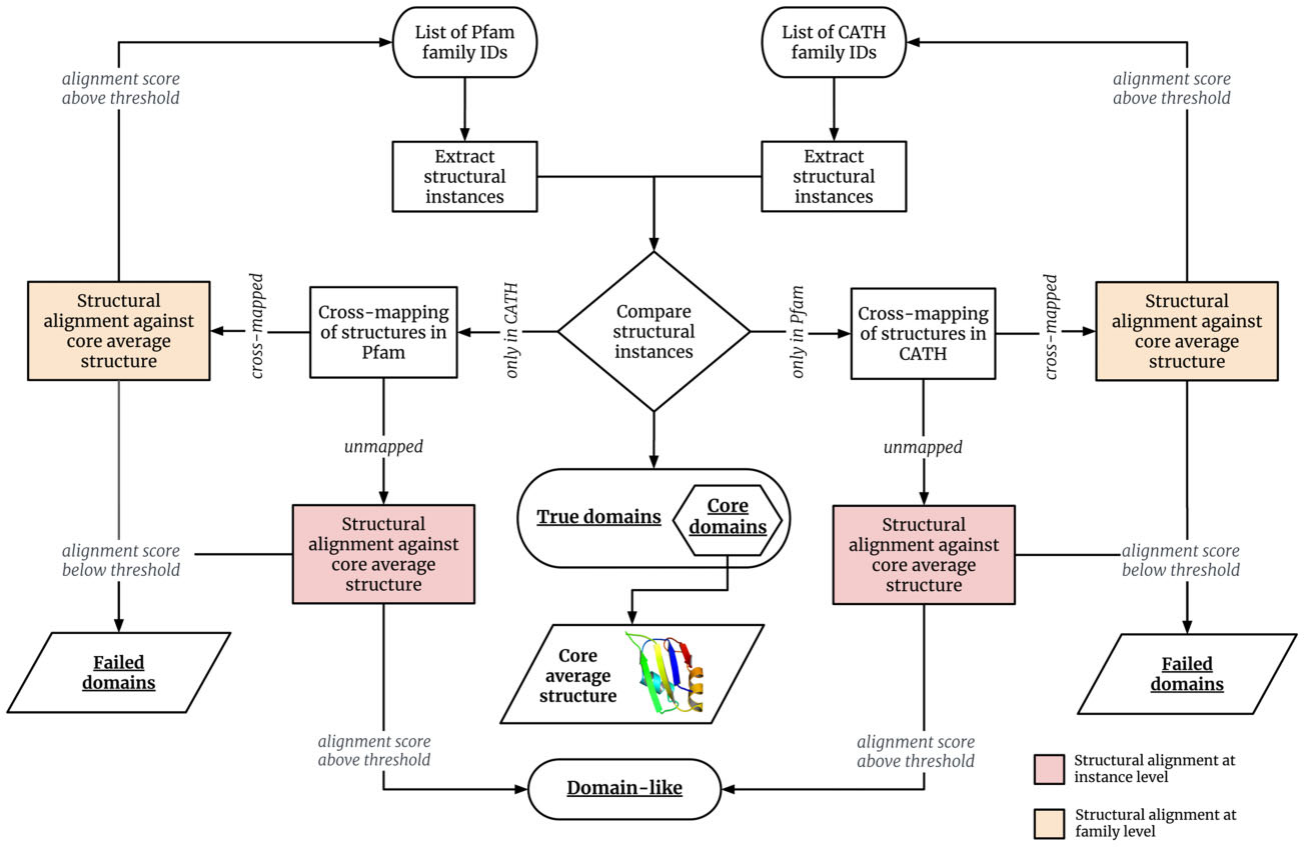
Conceptual model behind the CroMaSt workflow. The ‘core average structure’ computed at first iteration is used as a structural reference in all steps depicted as ‘structural alignment against core average structure’

More precisely our CroMaSt workflow is described in Figure 3. Our approach starts with a single domain family (or a list of domain families) representing the domain type of interest in each of Pfam and CATH domain databases. We then extract all StIs from these families and compare the lists obtained from each domain database. The instances common to both Pfam and CATH families are included in the ‘True’ domain list and named the ‘Core’ domain set at the first iteration of the workflow. An average structure is computed for the set of ‘Core’ domains and named ‘Core average structure’. The rest of the instances that are either specific to Pfam or specific to CATH are ‘cross-mapped’ in the other database to fetch possible additional CATH or Pfam (respectively) families for the same domain type. For all newly fetched families, an average structure is computed at the family level with the cross-mapped StIs. After verifying that the structural alignment of the new family members with the core average structure exceeds a certain quality threshold, the newly found family is added to the list of families at the beginning of the workflow and a new iteration is started. At each iteration, a certain number of domain StIs specific to each database remain un-mapped in the other database. For all un-mapped StIs corresponding to the same protein sequence, the average structure is computed at the sequence instance level. These averaged structures are checked by structural alignment with core average structure and classified as ‘Domain-like’ structures when the alignment score exceeds a given threshold. This iterative procedure is followed until no new family is found. Domain StIs that do not fall in any category (‘Core’, ‘True’ or ‘Domain-like’) are labeled as ‘Failed’ domains.

## 3 Methods

### 3.1 Selection of data sources

The files used in this study are as (i) the CATH domain description file—v4.3.0, (ii) from Pfam: the file with Pfam-A matches for each PDB chain—v35.0, (iii) from PDB (Bittrich *et al.*, 2022): the obsolete PDB entries information (accessed on June 1, 2023) and the 3D coordinates of PDB structures and (iv) the SIFTS mapping files (Dana *et al.*, 2019) (accessed at the time of the run). All data files can be downloaded by running a tool provided with the workflow that allows users to select the version of their choice for each source database. Only experimental PDB entries are referenced in CATH and Pfam.

### 3.2 Retrieve the domain StIs

The workflow starts with at least one domain family or superfamily of the studied domain type from each database. These families can be obtained from keyword or PDB ID search in each database. It is recommended to start with the most populated families of the studied domain type to get comprehensive results. There should be at least one common PDB structure between the starting families from each database, to be able to compute a reference structure for the studied domain type.

We define a domain ‘structural instance’ of a domain family as the part of the PDB chain that contains the domain region in a considered protein. This information consists of a PDB identifier, a chain identifier and the start and end residue positions. It can be extracted from Pfam and CATH raw files for each family member associated with one or more PDB entries. However, it is not easily compared between the two sources as domain StIs retrieved from the Pfam database have start and end residues numbered according to UniProt, while these residues are numbered according to PDB in the CATH database. Therefore, a residue-mapping step is required to generate a unified representation that follows the form *‘PDB_id, Chain_id, Domain_name or Domain_order_number, Family_id, PDB_start, PDB_end, UniProt_id, UniProt_start, UniProt_end’*. For example, a StI shared in Pfam and CATH has the following representation:

- ‘1CVJ,G,RRM_1,PF00076,13,83,P11940,13,83’ in Pfam, and
- ‘1CVJ,G,01,3.30.70.330,11,87,P11940,11,87’ in CATH.

This unified format facilitates identification of shared StIs between the two databases (see Section 3.3). Moreover, this format allows to save the start and end positions from the PDB chain in order to extract the 3D coordinates of the domain StIs for computing structural average and for structural alignment purpose. However, this format does not take into consideration discontinuous domains, which are handled by CATH but not Pfam (see 1AGRA01 in CATH and 1AGR in Pfam). Thus, the current implementation of CromaSt does not handle discontinuous domains.

**Figure 4.**
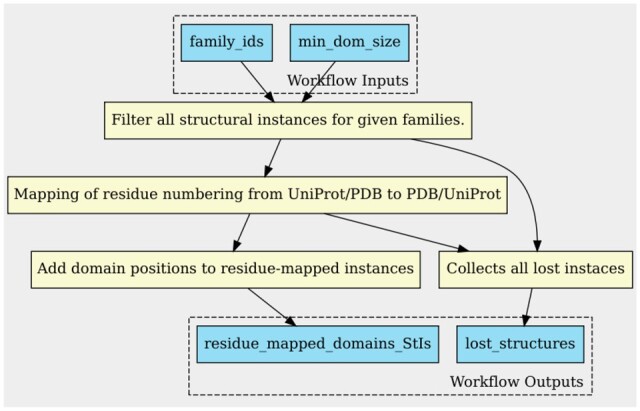
Process of filtering and residue-mapping of domain StIs (generated using cwltool)

The first step of the workflow consists of retrieving all StIs corresponding to the selected starting domain family IDs from each database. Domain length is used as a threshold to filter the domain StIs to avoid getting extremely short structures. Then, the start and end residue positions from the chain of retrieved StIs are mapped to their corresponding numbering on the associated PDB chain (for StIs derived from Pfam) or UniProt sequence (for StIs derived from CATH). The SIFTS resource is used for this residue-mapping step. The workflow also keeps track of the domain StIs for which the residue-mapping cannot be done (obsolete PDB entries or inconsistent mapping, see Supplementary Section C). The complete step for extracting the domain StIs is depicted in Figure 4.

### 3.3 Compare sets of domain StIs

At this stage, the CroMaSt workflow compares two sets of domain StIs (one from Pfam, one from CATH) using the unified StI representation and more precisely the *PDB_id, Chain_id, UniProt_start and UniProt_end* values. The workflow takes into account that the two databases can provide different lengths for the same domain instance, allowing for a difference between start (respectively end) residues for the same domain instance. By default, we use a maximum difference of 30 residues, but users can change this setting as needed. This step of the workflow results in three different sets: Common domain StIs, Domain StIs unique to Pfam and Domain StIs unique to CATH.

The common domain StIs obtained at the first iteration of the workflow are the ‘Core’ domains and are used to compute the core average structure of the domain type (Step 3.5.2). The domain StIs unique to each database must be ‘cross-mapped’ to the other database.

### 3.4 Cross-mapping of the unique domain StIs

Each domain StI unique to one database (CATH or Pfam) is ‘cross-mapped’ to the other database (Pfam or CATH, respectively). To do so, the PDB and chain IDs are used to query the domain description files downloaded from the data sources. When a hit is found, the start and end positions (from UniProt for Pfam database, and from PDB for CATH database) are checked with the same tolerated difference as in Step 3.3. If the cross-mapping is successful, the corresponding cross-mapped StI is created in order to be tested for the ‘True’ domain category by structural alignment (Steps 3.5.2 and 3.6). If the cross-mapping is not successful, the corresponding StI is also created in view of structural alignment and is stored as un-mapped. Some of these un-mapped StIs may result from the fact that the two domain databases rely on different releases of sequence databases. Nevertheless, un-mapped StIs will also undergo a structural alignment test (see Section 3.6.2) to avoid missing any instance of the domain type of interest.

In summary, this step provides three different sets of domain StIs as follows: Pfam StIs cross-mapped to new families in CATH, CATH StIs cross-mapped to new families in Pfam and Un-mapped StIs from both databases.

### 3.5 Computation of average structures

#### 3.5.1 Family-level and instance-level average structure

The coordinates for average structure are computed using an in-house python script after aligning a set of 3D coordinates (extracted from PDB entries) using the Kpax program (Ritchie, 2016). The resulting average structure only consists of the protein backbone without side-chains. When applied to a set of domain StIs, this computation could be biased toward the domain family members (defined by their UniProt sequence) having more StIs in the PDB. To avoid this, our workflow first computes ‘UniProt-instance-level’ averages from all StIs corresponding to the same domain in UniProt. Then, these ‘UniProt-instance-level’ average structures are used to compute the ‘family-level’ average structure as an ‘average of averages’.

#### 3.5.2 Core average structure

‘Core’ domain StIs are the common domain StIs (from Step 3.3) retrieved during the first iteration. Core average structure is computed using these ‘Core’ domain StIs as an average of ‘UniProt-instance-level’ averages, as described in Step 3.5.1. It should be noted here that the core average structure of a given domain type is dependent on the families selected for the first iteration of the workflow.

#### 3.5.3 Average structures for each cross-mapped families

An average structure is computed for each cross-mapped (newly found) family from all the unique domain StIs that are mapped to this family (Step 3.4). These average structures are computed at the ‘family-level’ as described in Step 3.5.1.

#### 3.5.4 Average structures for un-mapped domain StIs

Average structures are computed at the ‘UniProt-instance level’ for each un-mapped domain StI produced at Step 3.4.

### 3.6 Structural alignments

The core average structure of the domain obtained at Step 3.5.2 plays a crucial role in the structural alignments aimed at assigning the cross-mapped or un-mapped StIs to the studied domain type. The workflow considers the core average structure as the prototype of the studied domain type. For all structural alignments, the CroMaSt workflow uses Kpax as it provides a Gaussian-based Multiple Structural Alignments quality measure called ‘M-score’ (Ritchie, 2016), which circumvents the pitfalls of RMSD-based quality measures (Kufareva and Abagyan, 2012). This step outputs a *csv* file with all the scores provided by Kpax for each target structure. By default, the CroMaSt workflow uses ‘M-score’ as the alignment score and 0.6 as the score threshold to evaluate the alignments. Users can choose to evaluate the alignments using any other alignment score provided by Kpax (such as *K*-score, *J*-score, *T*-score and so on) with different thresholds. Depending on the domain type studied, users can run some tests and visualize some of the ‘True’ and ‘Failed’ StIs in order to make an educated guess of which threshold to use. For RRM domains, which are ∼90 amino acids long, we used the *M*-score with 0.6 threshold. It also worked nicely for one other domain type shown in Supplementary Section H.

#### 3.6.1 Structural alignments for average structures of cross-mapped families

Each average structure for cross-mapped families from Pfam and CATH (Step 3.5.3) is aligned against the core average structure using Kpax. If the average structure for a cross-mapped family passes the given threshold (for alignment score), the family identifier is added to the list of families for the next iteration of the workflow. In parallel, the StIs that were mapped to this new family are also kept for the next iteration in order to be recognized as ‘True’ domain StIs when comparing lists from the two domain databases. If the average structure for a cross-mapped family does not pass the threshold, this family and the corresponding cross-mapped StIs are labeled as ‘Failed’.

#### 3.6.2 Structural alignments for un-mapped average structures

The un-mapped average structures (from Step 3.5.4) are also aligned the same way as in Step 3.6.1. The StIs corresponding to average structures passing the threshold (for given score) are included in the list of ‘Domain-like’ StIs. The StIs corresponding to average structures failing to pass the threshold are considered as false positives and labeled as ‘Failed’.

### 3.7 Implementation

CroMaSt uses the Common Workflow Language (CWL) (Crusoe *et al.*, 2022) engine and the Conda package manager (to install the required dependencies). All the scripts are written in Python and wrapped in CWL as cwltools. Although the developers of CWL are working on adding the functionality for loop from quite some time now, CWL does not support the loops yet. As the nature of the CroMaSt workflow is iterative, a new parameter file is created at the end of each iteration, ready for the next iteration. This step updates certain inputs from string types to file types along with updating the most important input parameters of family identifiers (from Step 3.6.1). The family identifiers are updated based on the alignment scores of average structures for cross-mapped families. FAIR principles were followed while developing the workflow. As a result, the workflow can be found on WorkflowHub (Goble *et al.*, 2020) from doi: 10.48546/workflowhub.workflow.390.1. Details on how to use the CroMaSt workflow are provided on WorkflowHub and Git repository.

## 4 Results

To demonstrate the capacity of the CroMaSt workflow to distinguish between ‘True’ or ‘Domain-like’ and ‘Failed’ domain StIs with respect to a given domain type, we apply CroMaSt to the RRM domain type.

**Figure 5.**
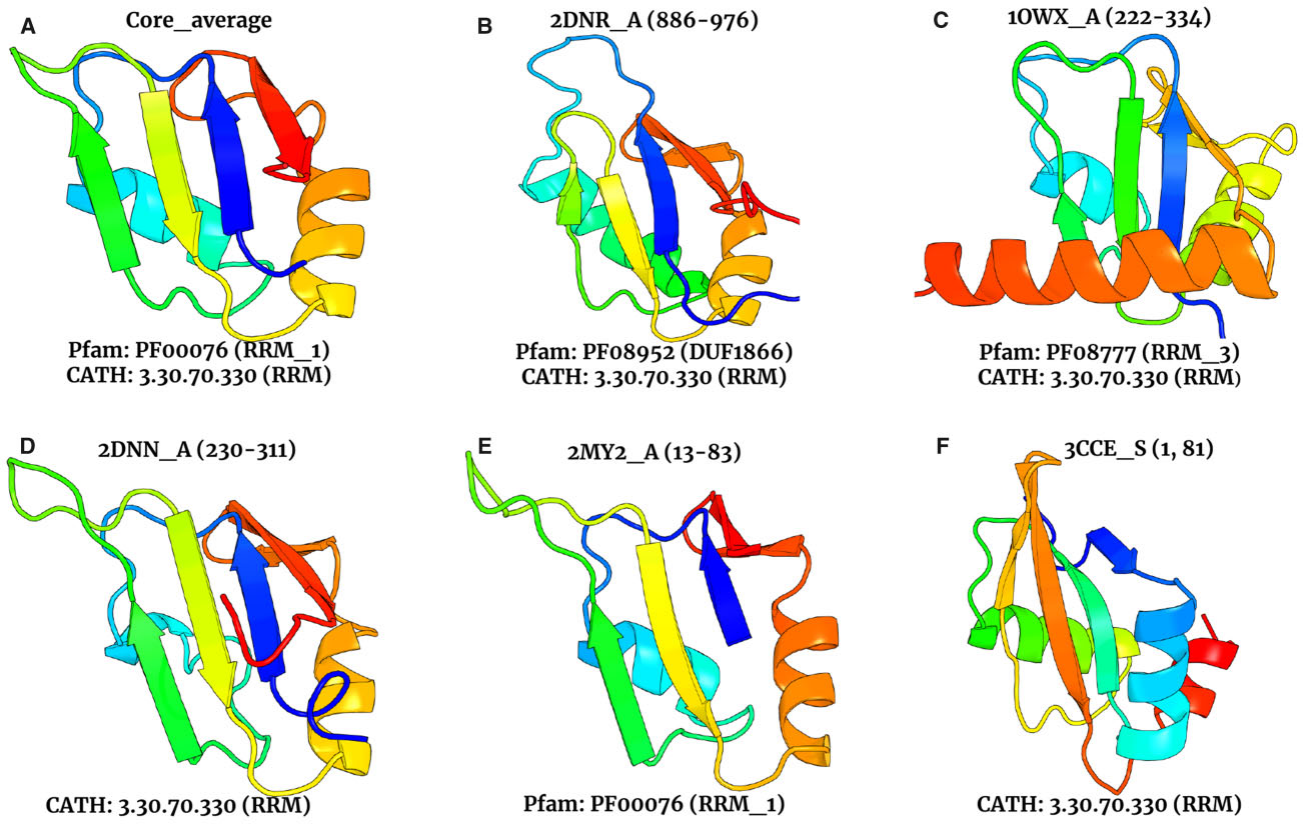
Examples of StIs for RRM domain type. (**A**) ‘Core average structure’ of RRM domain type. (**B and C**) ‘True’ domain StIs unique to CATH at first iteration and successfully mapped to two different Pfam families. (**D and E**) ‘Domain-like’ StIs from CATH and Pfam, respectively. (**F**) ‘Failed’ domain StI from CATH 3.30.70.330 superfamily

We initiate the CroMaSt workflow with families PF00076 (RRM_1) from Pfam and superfamily 3.30.70.330 (RRM domain) from CATH. Table 2 shows the different results obtained at each step of the workflow. A total of 1334 domain StIs were extracted from RRM_1 Pfam family of which 1 was inconsistent, whereas 1527 domain StIs were extracted from CATH, of which 316 were obsolete and 7 inconsistent. Then, the 1333 and 1204 domain StIs from Pfam and CATH, respectively, were residue-mapped (Fig. 4). Out of all these residue-mapped domain StIs, 883 are shared between Pfam and CATH. Thus, 883 StIs constitute the ‘Core’ domain StIs, and are also included in the list of ‘True’ domain StIs. Core average structure (Fig. 5A) for RRM domain type was computed using these 883 StIs. From the remaining StIs (450 unique to Pfam and 321 unique to CATH), only 243 StIs from CATH were successfully cross-mapped to a total of 17 different Pfam families. Thus, ‘family-level’ average structures were computed for these 17 newly found Pfam families using the cross-mapped StIs. After aligning these average structures against the core average structure, 14 of them (corresponding to 79 cross-mapped StIs) passed the threshold (*M*-score ≥0.6) allowing to include these families at the beginning of the next iteration. The remaining three families and their corresponding StIs (164) did not pass the structural alignment step (*M*-score < 0.6) and were considered as ‘Failed’ domain families and StIs (see Supplementary Section D for more details on the results of the structural alignment step for cross-mapped StIs). Thus, only 79 StIs from the 243 CATH StIs cross-mapped to Pfam and 14 new Pfam families were kept for the next iteration.

**Table 2.** Results from each step of a CroMaSt workflow execution, starting with Pfam family PF00076 (RRM_1) and CATH superfamily 3.30.70.330 (RRM domain)

Steps	Iteration 1		Iteration 2	
	Pfam	CATH	Pfam	CATH
Starting families	1	1	14	0
StI filtered on domain length	1334	1527	100	79^a^
Obsolete and inconsistent entries	1	323	0	0
Residue-mapped StIs	1333	1204	100	—
Common StIs (‘Core’ & ‘True’)	883	883	79	79
Remaining StIs (not common)	450	321	21	0
Cross-mapped StIs	0	243	0	0
Properly aligned at family level (‘True’)	—	79^b^	—	—
Not properly aligned at family level (‘Failed’)	—	164^c^	—	—
Un-mapped StIs	450	78	21	0
Properly aligned at instance level (‘Domain-like’)	443	78	20	—
Not properly aligned at instance level (‘Failed’)	7	0	1	—
Total ‘Failed’ StIs	7	164^c^	1	0
‘Failed’ domain families	3	0	0	0
New domain families for RRM domain type	14	0	0	0

a CATH StIs from the previous iteration, successfully cross-mapped and properly aligned at the family level.

b Corresponding to 14 Pfam families assessed as RRM domain type.

c Corresponding to three Pfam families not assessed as RRM domain type.

The average structures were computed at the ‘UniProt-instance-level’ for all un-mapped StIs from Pfam (450) and CATH (78). After structural alignment against the core average structure, only seven StIs from Pfam failed to pass the threshold. Thus, all remaining StIs (443 from Pfam, and 78 from CATH) are qualified as ‘Domain-like’ StIs.

In summary, the first iteration resulted in a total of 883 ‘Core’, 521 ‘Domain-like’ and 171 ‘Failed’ domain StIs, with 14 Pfam families and 79 CATH StIs ready for the next iteration.

The second iteration started with the 14 Pfam families and 79 StIs from CATH. A total of 100 StIs were extracted from the 14 Pfam families with no inconsistent or obsolete entry. The two sets shared 79 StIs (‘True’ domain StIs) and the other 21 StIs from Pfam remained un-mapped in CATH. Nearly all of them (20/21) passed the alignment threshold leading to 20 ‘Domain-like’ StIs and 1 ‘Failed’ domain StI. Thus, at the end of the second iteration, no new family was found, hindering any further iteration of the workflow.

The CroMaSt workflow keeps track of all obsolete and inconsistent domain StIs, which are detected mostly at the residue-mapping step based on SIFTS. To illustrate that, we listed the inconsistent StIs in Supplementary Section C. For example, the start and end residues of domain StI present in PDB ID ‘2KU7’ (chain A) are mapped to two different UniProt IDs: Q03164 and Q9UNP9 in SIFTS. Such inconsistencies deserve careful attention and manual curation that is beyond the scope of this work. Nevertheless, we considered it relevant to make this list of inconsistencies available to potential curators as an output of CroMaSt.

In summary, the CroMaSt workflow, initialized with Pfam PF00076 and CATH 3.30.70.330 domain families, identified 962 ‘True’ domain StIs (among which 883 are ‘Core’), 541 ‘Domain-like’ and 172 ‘Failed’ domain StIs with respect to the RRM domain type. In terms of domain families, the CroMaSt workflow explored a total of 19 families (18 from Pfam and 1 from CATH, including the starting families) and 16 of them (15 from Pfam and 1 from CATH) qualified for the RRM domain type (Table 3). Interestingly, all 15 Pfam families are members of the RRM clan CL0221 in Pfam whereas the 3 Pfam families which did not pass the structural alignment step are not. Thus, in this setting, CroMaSt did not detect any new families to add to this clan. In Table 3, we see that CroMaSt detected ‘True’ domain StIs in one Domains of Unknown Function (DUF) Pfam family, DUF1866. This family was already member of the RRM clan, but the CroMaSt results show that it can now be associated to an RNA-binding function with greater confidence. We also mapped the 15 Pfam families qualified as RRM domain type to structural domain databases other than CATH, namely ECOD and SCOP. This process detected incompleteness in SCOP but ECOD was well aligned with Pfam (see Supplementary Section E and Supplementary Table S2).

**Table 3.** List of 19 domain families explored by the CroMaSt workflow

Family IDs	Family name	Database	Passed alignment threshold?
PF00076	RRM_1	Pfam	NA^a^
PF00276	Ribosomal_L23	Pfam	No
PF00511	PPV_E2_C	Pfam	No
PF01282	Ribosomal_S24e	Pfam	No
PF03467	Smg4_UPF3	Pfam	Yes
PF03880	DbpA	Pfam	Yes
PF04847	Calcipressin	Pfam	Yes
PF05172	Nup35_RRM	Pfam	Yes
PF08675	RNA_bind	Pfam	Yes
PF08777	RRM_3	Pfam	Yes
PF08952	DUF1866	Pfam	Yes
PF09162	Tap-RNA_bind	Pfam	Yes
PF11608	MARF1_RRM1	Pfam	Yes
PF11835	RRM_8	Pfam	Yes
PF13893	RRM_5	Pfam	Yes
PF16367	RRM_7	Pfam	Yes
PF16842	RRM_occluded	Pfam	Yes
PF17774	YlmH_RBD	Pfam	Yes
3.30.70.330	RRM (RNA recognition motif) domain	CATH	NA^a^

a Starting domain families from Pfam and CATH. NA, not applicable.

In total, this run of CroMast workflow lasted 68.9 min on a machine equipped with eight 2.40 GHz Intel(R) Xeon(R) Silver 4214R processor without any prior downloads of PDB and SIFTS entry files.

Figure 5 illustrates the diversity of ‘True’ and ‘Domain-like’ StIs identified with CroMaSt. The core average structure of RRM domain type calculated by CroMaSt with the ‘Core’ domain StIs is shown in panel A. In panels B and C, the ‘True’ domain examples have some extensions at the C-terminal end of the typical RRM topology in the form of a β sheet or an α helix, respectively. These extensions of the RRM domain type have been studied previously and are in good agreement with the variations described by Maris *et al.* (2005). The ‘Domain-like’ examples shown in panels D and E are also consistent with variations described earlier. In particular, the one on panel D has a β sheet within the loop5 that is also found in many domain StIs from Pfam family PF00076 (RRM_1) and CATH superfamily 3.30.70.330 (RRM). In panel F, the ‘Failed’ domain example has a different topology from the typical RRM domain type. Other examples of ‘Failed’ StIs are shown in Supplementary Figures S1 and S2.

## 5 Discussion

The multiplicity of biological databases and the lack of systematic cross-references between them lead to important issues in data integration and consistency. Protein domain databases are no exception. Here, we show the way to increase interoperability between protein domain databases using a cross-mapping approach. Our CroMaSt method constitutes a systematic, reproducible and automated solution to retrieve domain StIs corresponding to a certain domain type of interest for the user. Along with this, CroMaSt also points out some irregularities in the databases.

It should be noted that the results returned by CroMaSt are determined by the starting domain families used as input. In fact, the set of ‘Core’ domain StIs is dependent on the starting domain families. This influences the computation of the ‘core average structure’ of the domain type and in turn all other results. This strong dependency of CroMaSt on input domain families has at least two practical consequences. Firstly, it is recommended to start the workflow with the two most populated domain families in order to get the most exhaustive results. It is also possible to start with more than one family from one or both databases. Second, this feature can be used to explore particular domain families within a given domain type in order to characterize possible sub-type core average structures.

In the setting described in this article, we found only 15 from the 33 Pfam families currently grouped in the Pfam RRM clan CL0221 (Supplementary Section F). There are various explanations for the 18 missing families. The first one is that five Pfam families are devoid of any StI and cannot be assessed by CroMaSt for this reason. For the 13 other missing families, one reason may be that the list of CATH StIs is incomplete as it does not rely on the same UniProtKB releases as Pfam. Thus, it is possible that there were no StI unique to CATH to be cross-mapped to any of these 13 Pfam families. The other reason is that the 80 StIs present in these 13 Pfam families may be present in CATH but in other superfamilies than the 3.30.70.330 one used as input to CroMaSt. We investigated this last explanation by giving as inputs to the CroMaSt workflow the same PF00076 and CATH 3.30.70.330 domain families plus the 13 Pfam families missing from the first run. As expected, the 80 StIs corresponding to these 13 Pfam families were collected as Unique to Pfam but they detected four new CATH superfamilies at the cross-mapping step (see results in Supplementary Section G and Supplementary Tables S3 and S4).

The structures predicted by AlphaFold2 (Jumper *et al.*, 2021) for all UniProt sequences can be used to increase the power of the CroMaSt workflow. In particular, Alphafold2 models would be useful to inspect domain families that are devoid of any experimental StI to date (e.g. the five Pfam families from RRM clan mentioned above). Such families would be enriched with at least as many AlphaFold2 StIs as UniProt family members. However, in its current implementation, CroMaSt can only consider AlphaFold2 predicted 3D models if they are already included in the domain database classifications. Moreover, the computation load would increase a lot for the CroMaSt workflow and some adaptation would be required to allow more distributed or parallel computing.

The CroMaSt workflow can be easily applied to any other domain type different from RRMs by providing appropriate family identifiers as inputs to the workflow. Nevertheless, as mentioned above, discontinuous domains are not yet handled by CroMaSt. Expert knowledge of the domain type of interest is useful to start the workflow with correct families and to inspect the structural alignments performed by the CroMaSt workflow. The allowed difference in size between domain boundaries in each database, the type of alignment score and the threshold value can be changed depending on the structural versatility of the domain type. If the user is unsure about the threshold, running the first iteration of the CroMaSt workflow with the default threshold is recommended. After the first iteration, all the 3D alignment results are available for manual inspection in order to tune the parameters if needed. We provide two other examples of CroMaSt usage in Supplementary Section H: one for the ‘Cadherin’ domain type and one for the ‘Zinc Finger’ domain type that were run with the default alignment threshold. For the first example the results are satisfactory, but for the second example analysis of the failed StIs suggests that the threshold should be lower likely because of the very short domain length (∼25 amino acids) of this domain type.

The CroMaSt workflow allows individual users, studying particular domain types, to contribute to the curation of Pfam and CATH protein domain databases. For instance, they can detect irregularities in domain boundaries or annotations within databases and point to discrepancies among databases. They can also suggest a function for domain families lacking any annotation (e.g. the DUF domains in Pfam) and highlight StIs wrongly assigned to a domain family or domain type. Two examples illustrate this. In Pfam, we observed that one ‘Failed’ domain StI from RRM_1 domain family (PF00076), derived from the 5OPT_h PDB entry, has been assigned by Pfam erroneous domain boundaries resulting in the loss of β1 and β4 at N-terminal and C-terminal ends of the StI. In CATH, CroMaSt detected a member of the CATH RRM superfamily 3.30.70.330, having the same topology as RRM domain type but lacking the RRM specific sequence signatures (RNP1 and RNP2), thanks to the fact that this domain was cross-mapped to the PPV_E2_C (PF00511) domain family in Pfam, which did not pass the structural alignment step (see Supplementary Fig. S2). In general, interested users should inspect ‘Failed’ StIs in order to detect similar situations.

Currently, CroMaSt takes Pfam and CATH as sources for domain databases. However, CroMaSt can be expanded to include other domain databases as well. As the cross-mapping step can lead to many un-mapped StIs because the compared databases do not use the same version of UniProtKB, CroMaSt should in future take advantage of the integrated InterPro domain family resource, which runs all the domain models on the same version of UniProtKB.

## 6 Conclusion

CroMaSt is a fully automated workflow that assesses the assignment of protein domains to a given domain type thanks to cross-mapping between CATH and Pfam domain databases and to 3D structural alignment. The multiple structural alignments provided by CroMaSt can provide new information about conserved and variable residues, loops or SSEs, for evolutionary studies of a given domain type. In the case of RRM domain type, such information was exploited recently to decipher the RNA recognition code for the canonical binding mode to RRM domains (Martinez *et al.*, 2023). More generally, we believe that CroMaSt results can be readily used in protein design, for building synthetic proteins carrying properties of the desired domain type.

## Supplementary Material

vbad081_Supplementary_Data

## Data Availability

The data underlying this article are available in Workflowhub at https://doi.org/10.48546/workflowhub.workflow.390.2.
