# Supplementary material for: CroMaSt: a workflow for assessing protein domain classification by cross-mapping of structural instances between domain databases and structural alignment
Source: Bioinform Adv. 2023 Jun 27;3(1):vbad081. doi: 10.1093/bioadv/vbad081 (PMC10329740; doi:10.1093/bioadv/vbad081)
Supplement: vbad081_Supplementary_Data [file vbad081_supplementary_data.pdf]

Supplementary Information
CroMaSt: A workflow for assessing protein domain classification by cross-mapping of structural instances between domain databases and structural alignment

Hrishikesh Dhondge, Isaure Chauvot de Beauchêne, and Marie-Dominique Devignes *

June 2023

*This work was supported by funding from the Marie Skłodowska-Curie Innovative Training Network (MSCA-ITN) RNAct [813239].
The computational support was funded by Contrat Plan État Région, Innovations Technologiques, Modélisation & Médecine Personnalisée (CPER ITM2P); and Fonds Européen de Développement Régional (FEDER).

A. Review of existing domain databases

Among sequence-based domain databases, Pfam is certainly one of the most comprehensive resources [Mistry et al., 2021]. Seed alignments for a representative set of sequences are manually curated using structural information, whenever available. Then, an HMM (Hidden Markov Model) profile is built based on the seed alignment which is then used to retrieve a full set of sequences matching this domain from the UniProt reference proteomes, thus producing the Pfam entry full alignment. Sets of Pfam entries that are thought to be evolutionarily related are grouped together into clans. This grouping is based on sequence similarity, structural similarity, functional similarity and/or profile-profile comparisons. Since October 5, 2022 the Pfam website is redirected to InterPro as it was decommissioned in January 2023. However the Pfam project continues and the core database will be maintained on the same principles as before [Paysan-Lafosse, 2022].

Structure-based domain databases are best represented by the SCOP (Structural Classification of Proteins) [Andreeva et al., 2020], CATH (Class, Architecture, Topology and Homology) [Sillitoe et al., 2021] and ECOD (Evolutionary Classification Of protein Domains) [Cheng et al., 2014] databases. The SCOP database aims to provide a detailed and comprehensive description of the structural and evolutionary relationships between proteins whose three-dimensional structure is known and deposited in the PDB. The classification of proteins in SCOP has been constructed mainly manually by visual inspection and analysis. In SCOP, entries are protein domains identified in PDB structures and organized into families and superfamilies and finally into structural folds and classes reflecting their secondary structure content. Domain boundaries are provided at both family and superfamily levels as the evolutionary relationships can sometimes span regions of different size between closely related (family level) and more distantly related (superfamily level) proteins. In brief, the family domain boundaries can define conserved multi-domain regions whereas the superfamily domains span over the individual domains. The relationships between the SCOP and Pfam databases are complex because of differences in boundaries and members of the families. However, curated cross-references are indicated at the family level from SCOP to Pfam database in many cases.

The CATH project has developed a semi automatic procedure to split 3D structures into the constituent domains defined as semi-independently folding globular units. These domains are then clustered into homologous superfamilies based on evolutionary relationships. The sequences of the CATH structural domains are then used to build HMM profiles in order to identify the domains in UniProt protein sequences for which no 3D structure is available. This effort is shared with the sister resource Gene3D. The lowest level of CATH is H for Homologous superfamilies and CATH provides structural superpositions of all representative domains of a superfamily. However superfamilies can be sub-divided in functionally coherent groups named Functional Families (FunFams). Recently CATH has created an additional class for non globular domains.

The ECOD is a hierarchical classification consisting of five levels: architecture (A), possible homology (X), homology (H), topology (T), and family (F). The entire database is first divided into architectures (A) based on secondary structure element (SSE) composition and overall shape (for example, alpha bundles and beta sandwiches). Then, possible homology (X) groups domains where some evidence exists to demonstrate homology but further evidence is needed. At the homology (H) level, ECOD groups domains that are descended from a common ancestor as indicated by significant sequence-structure scores, functional similarity, opinions in literature and in SCOP. The topology (T) level groups domains that have similar arrangements of and connections between secondary structural elements. At the bottom-most level, the family (F) level groups domains that have significant sequence similarity primarily based on Pfam. ECOD employs an automatic software pipeline to classify newly released structures from PDB. Proteins that cannot be classified confidently and completely by automated methods are manually curated.

The third category of domain databases encompasses integrated databases such as InterPro and CDD (Conserved Domain Database). InterPro [Blum et al., 2021] is an integrated resource of predictive models or 'signatures' representing protein domains, families, regions, repeats and sites from major protein signature databases including CATH-Gene3D, HAMAP, PANTHER, Pfam, PIRSF, PRINTS, PROSITE, SMART, SUPERFAMILY and TIGRFAMs. Thus, InterPro aims to combine the individual strength of each protein domain database without building domain models itself. Quality control is performed at InterPro when integrating new signatures by checking whether such signatures generate false positive matches. Hierarchical relationships are identified between InterPro entries to represent subfamilies displaying specific functions within larger families, or specific subclasses within certain classes of domains. The InterProScan software regularly calculates InterPro signature matches to UniProtKB.

The CDD database [Lu et al., 2020] is a protein annotation resource that consists of a collection of well-annotated multiple sequence alignment models for domains and full-length proteins obtained from both NCBI projects (NCBI Protein Clusters collection, NCBIfam, CDD itself) and external sources (Pfam, SMART, COG, TIGRFAMs). These models are available as position-specific score matrices (PSSMs) for fast identification of conserved domains in protein sequences via RPS-BLAST (Reversed Position Specific BLAST). The NCBIfam curated domains use 3D structure information to define domain boundaries explicitly and provide insights into sequence/structure/function relationships. CDD shares domain models with InterPro and contributes to enlarge InterPro with specific subfamilies.

B. Inconsistencies in Pfam and CATH for RRM domain type

Inconsistencies in and between domain databases regarding RRM domain families appear with very simple searches. Inconsistencies are at the level of the databases, inside a database (ex : RRM families outside the RRM clan) and between databases (16 Pfam families versus 2 CATH superfamilies). Firstly, there are 16 Pfam families with RRM in their name: MARF1_RRM1 (PF11608), Nup35_RRM (PF05172), Nup35_RRM_2 (PF14605), RRM (PF10378), RRM_1 (PF00076), RRM_2 (PF04059), RRM_3 (PF08777), RRM_4 (PF10598), RRM_5 (PF13893), RRM_7 (PF16367), RRM_8 (PF11835), RRM_9 (PF18444), RRM_DME (PF15628), RRM_occluded (PF16842), RRM_Rrp7 (PF17799), xRRM (PF19977). In addition, Pfam also has an RRM clan (CL0221) with a total of 33 Pfam families including only 13 of the above-mentioned families with RRM in their name (see suppl

Table S3). In particular, the RRM clan does not include RRM (PF10378), RRM_4 (PF10598) and RRM_DME (PF15628). These 3 Pfam families do not belong to any clan in Pfam.

In CATH, 2 superfamilies have RRM keyword in their name: 3.30.70.330 ‘RRM (RNA recognition motif) domain’, 3.30.70.1820 ‘L1 transposable element, RRM domain’. When the CATH database was searched using keyword ‘RRM’, several matching CATH domains were retrieved apart from these two CATH superfamilies. Some of those hits are as follows: 3m4xA01 from Superfamily 3.30.70.3130[1], 3m4xA02 from Superfamily 3.40.50.150, 3m4xA03 from Superfamily 2.30.130.60, 3dxbA01 from Superfamily 3.40.30.10.

C. List of inconsistent structural instances

There are a total of 324 StIs from Pfam and CATH considered as obsolete and inconsistent entries by CroMaSt, of which 8 are inconsistent and the remaining are obsolete entries.

Format of these structural instance entries differ slightly for Pfam and CATH.

- Pfam inconsistent and obsolete entries: “PDB_id,Chain,Fam_name,Fam_id,UNP_id,UNP_start,UNP_end”
- CATH inconsistent entries: “ PDB_id,Chain,Domain_position,Fam_id,PDB_start,PDB_end”
- CATH obsolete entries: “ PDB_idChainDomain_position,Fam_id,PDB_start,PDB_end”

Below is the list of the 8 inconsistent domain StIs detected in CroMaSt run1 (Main paper):

1. “5OSG,h,RRM_1,PF00076,E9BNI3,71,119”,
2. “2KU7,A,00,3.30.70.330,1,140”,
3. “3DXB,F,02,3.30.70.330,452,556”,
4. “3DXB,G,02,3.30.70.330,452,556”,
5. “3DXB,D,02,3.30.70.330,452,556”,
6. “3DXB,B,02,3.30.70.330,452,556”,
7. “3DXB,A,02,3.30.70.330,452,556”,
8. “4V19,X,00,3.30.70.330,2,150”

The domain StI with PDB ID ‘5OSG’ (chain h) is associated with UniProt ID ‘A0A3Q8IT45’ in PDB and with UniProt ID ‘E9BNI3’ in Pfam. This is because UniProtKB has merged ‘E9BNI3’ into ‘A0A3Q8IT45’ in a recent update (release 2023_02).
Regarding the domain StI present in PDB ID ‘2KU7’ (chain A), the start and end residues from the PDB entry are mapped in SIFTS to two different UniProt IDs: Q03164 and Q9UNP9. The situation is the same for all domain StIs in PDB ID ‘3DXB’, the start and end residues map to UniProt IDs, P0AA27 and Q9UHX1, respectively. Finally, the domain StI in PDB ID ‘4V19’ (chain X) has no annotation for any UniProt entry in SIFTS.

The complete list of obsolete domain StIs can be found in the Results archive of CroMaSt run1.

D. Structural alignment step at family level

All the cross-mapped structural instances (StIs) are first averaged into domain instances (UniProt-instance-level) followed by averaging all the domain instances together, resulting in the average structure at family level (See Methods section). All these average structures at family level are aligned against the core average structure using Kpax, and based on the alignment score the families are either added at the beginning of next iteration or discarded. Table S1 shows the excerpt from the alignment result file.

The three families failing to pass the given threshold (Mscore < 0.6) are Ribosomal_L23 (PF00276), PPV_E2_C (PF00511), and Ribosomal_S24e (PF01282). The average structures for these three families are shown in Figure S1. The topology (order of secondary structure elements) for all these structures are mentioned below -

1. Core average structure of RRM domain type - $\beta1$ $\alpha1$ $\beta2$ $\beta3$ $\alpha2$ $\beta4$
2. Ribosomal_L23 (PF00276) - $\alpha1$ $\beta1$ $\alpha2$ $\beta2$ $\beta3$
3. PPV_E2_C (PF00511) - $\beta1$ $\alpha1$ $\beta2$ $\beta3$ $\alpha2$ $\beta4$
4. Ribosomal_S24e (PF01282) - $\beta1$ $\beta2$ $\alpha1$ $\beta3$ $\beta4$

The topology of ‘core average structure’ for RRM and ‘PPV_E2_C’ domain types are similar, thus to confirm we visualized the structural alignment of PPV_E2_C with core average domain (Figure S2). RNP regions are highlighted (RNP1: Green, RNP2: Blue) in the sequence alignment (Figure S2 C.) showing the difference between these sequences (structures). The sequence from RNP regions of ‘PPV_E2_C’ (PF00511_avgStruct_core_avgStruct.pdb) does not match with the RNP sequence from core average structure (core_avgStruct_query.pdb), moreover ‘PPV_E2_C’ structure lacks aromatic residues in this region, that can form stacking interactions with nucleotides.

A total of 42 StIs were used to compute the average structure at the family-level for PPV_E2_C (PF00511). All of these StIs are originally from CATH StIs cross-mapped to PPV_E2_C (PF00511) Pfam family. All the StIs used to compute the average structure at family level can be found in the Results archive of CroMaSt run1.

E. Mapping of CroMaSt results to other structure-based domain databases

CroMaSt uses the cross-mapping approach for individual StI between Pfam and CATH. Although the results from CroMaSt covers both sequence (from Pfam) and structural (from CATH) features, we wanted to compare the results with other structure-based classifications, i.e., ECOD and SCOP. This comparison can be done at different levels (family and instance-level), but it is an extensive and time-consuming procedure. Thus, we randomly selected a StI from each Pfam family (with at least 1 StI in list of ‘True’ domain StIs) and cross-mapped these StIs to families in ECOD and SCOP. Table S2 shows StIs from Pfam and the cross-mapped families in ECOD and SCOP, respectively.

SCOP does not have a family exclusively named as ‘RRM’ or ‘RNA Recognition Motif’, but the ‘Canonical RBD’ family of SCOP can be cross-referenced to the ‘PF00076’ (RRM_1) of Pfam. The ‘Canonical RBD’ family classifies under the superfamily ‘RNA-binding domain, RBD’ in SCOP. All of the SCOP families from Table S2 are classified under the superfamily ‘RNA-binding domain, RBD’.

In ECOD, the family (F) level classification for domains is primarily based on Pfam domains having significant sequence similarity. Thus, the family naming convention is similar to the Pfam. All the ECOD families from Table S2 are classified under the same topology (T) - ‘RNA-binding domain, RBD’.

[1] merged with 3.30.70.1170: Sun protein; domain 3

Table S1. Structural alignment results of cross-mapped StIs using average stuctures at family level against the core average structure of the RRM domain type

Query In	Target In	Kscore	Mscore	Tscore	Ncover	Naligned	RMSD-aligned (in Å)
core_avgStruct	PF13893_avgStruct	59.01	0.9265	0.9257	77	74	1.04
core_avgStruct	PF11835_avgStruct	50.98	0.8603	0.8693	81	73	1.43
core_avgStruct	PF16367_avgStruct	49.39	0.7997	0.8040	97	72	1.66
core_avgStruct	PF04847_avgStruct	40.26	0.7976	0.7980	84	70	1.52
core_avgStruct	PF05172_avgStruct	47.64	0.7872	0.7872	76	69	1.52
core_avgStruct	PF16842_avgStruct	45.36	0.7852	0.7986	82	72	1.87
core_avgStruct	PF11608_avgStruct	45.65	0.7843	0.7855	78	68	1.72
core_avgStruct	PF03467_avgStruct	47.47	0.7757	0.7889	97	75	1.98
core_avgStruct	PF08952_avgStruct	47.62	0.7618	0.7713	90	69	1.77
core_avgStruct	PF03880_avgStruct	41.26	0.7557	0.7513	83	67	1.62
core_avgStruct	PF08675_avgStruct	35.01	0.7261	0.7281	109	65	1.62
core_avgStruct	PF17774_avgStruct	34.10	0.6756	0.6876	85	66	1.99
core_avgStruct	PF09162_avgStruct	39.94	0.6522	0.6738	82	69	2.31
core_avgStruct	PF08777_avgStruct	35.57	0.6184	0.6480	88	69	2.47
core_avgStruct	PF00276_avgStruct	35.14	0.5604	0.5648	96	50	1.76
core_avgStruct	PF00511_avgStruct	39.73	0.5360	0.5831	88	64	2.70
core_avgStruct	PF01282_avgStruct	26.57	0.4517	0.4584	70	43	1.96

*The complete file for these alignment results can be found in the Results archive of CroMaSt run1.

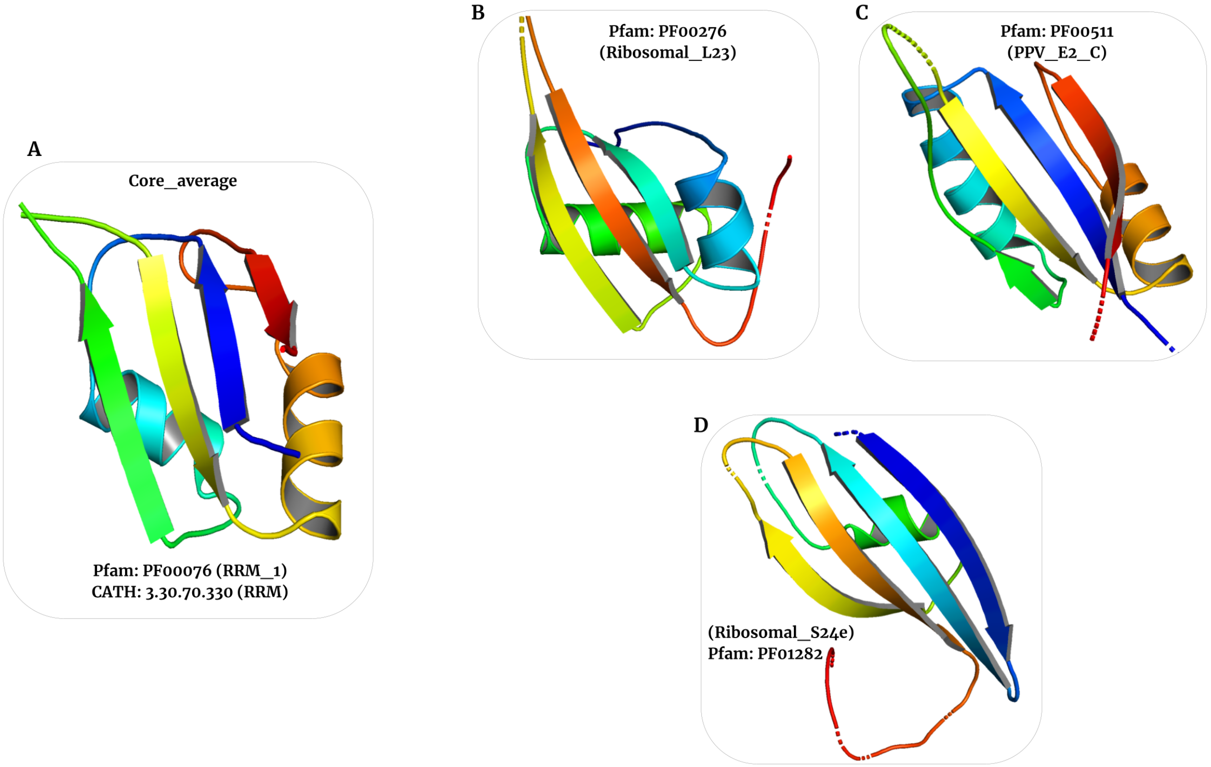

Fig. S1. A) Core average domain structure B) Average structure at family level for Ribosomal_L23 (PF00276) Pfam family C) Average structure at family level for PPV_E2_C (PF00511) Pfam family D) Average structure at family level for Ribosomal_S24e (PF01282) Pfam family.

In CATH, all these StIs from Table S2 are from superfamily 3.30.70.330 (RRM domain).

Table S2 contains only StIs from the list of 'True' domain StIs from CroMaSt. Three of these StIs [2E5I_A (208, 293),

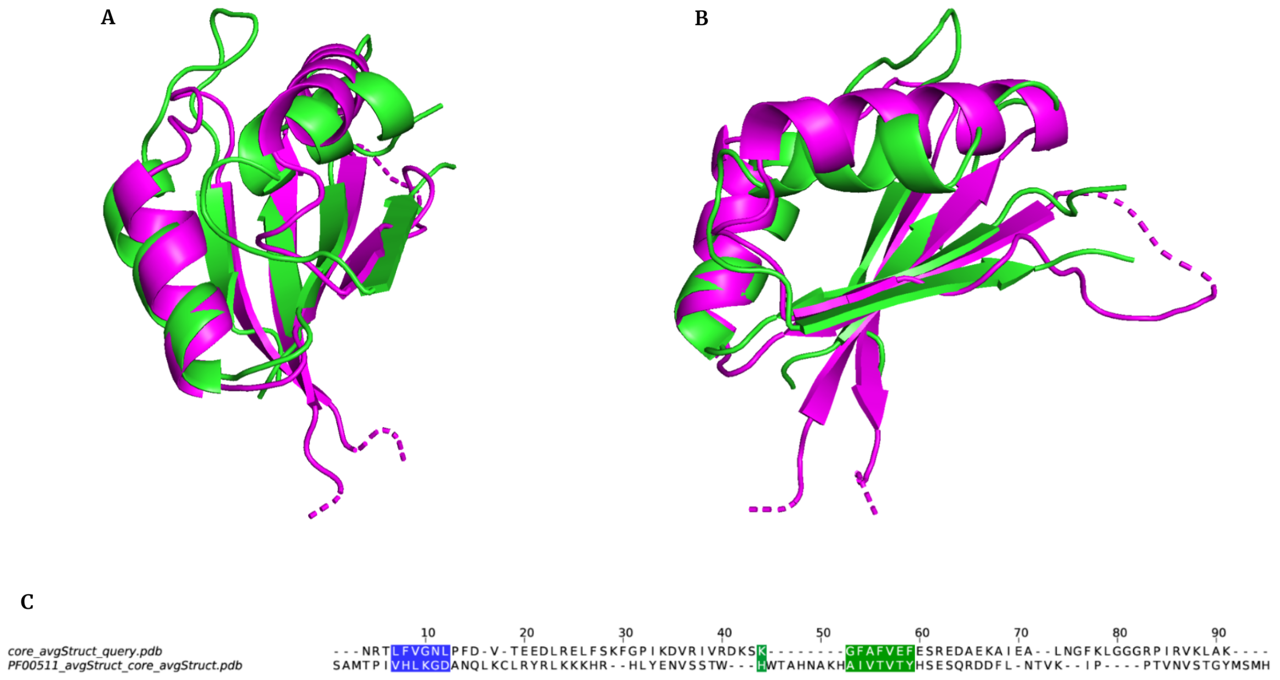

Fig. S2. Alignment of PPV_E2_C (PF00511) average structure (magenta) against RRM core average structure (green). A. and B. Two different views for the structural alignment. C. Fasta alignment resulting from structural alignment; Region highlighted in blue represents the RNP2 signature sequence and in green the RNP1 one.

Table S2. Cross-referencing of representative StIs from 'True' Pfam families in ECOD and SCOP databases

Pfam Family Name (Family Ids)	Representative StI	Family in ECOD	Family in SCOP2
RRM_1 (PF00076)	1B7F_A (127, 197)	RRM_1_2 (EF21352)	Canonical RBD
Smg4_UPF3 (PF03467)	1UW4_A (50, 140)	Smg4_UPF3 (EF12679)	Smg-4/UPF3-like
DbpA (PF03880)	2G0C_A (405, 476)	DbpA (EF02236)	DbpA RNA-binding domain-like
Calcipressin (PF04847)	1WEY_A (15, 98)	Calcipressin (EF01259)	Canonical RBD
Nup35_RRM (PF05172)	3P3D_A (266, 363)	Nup35_RRM (EF10021)	Canonical RBD
RNA_bind (PF08675)	3CTR_A (445, 514)	RNA_bind (EF12083)	Canonical RBD
RRM_3 (PF08777)	1OWX_A (231, 334)	RRM_3 (EF12206)	Canonical RBD
DUF1866 (PF08952)	2DNR_A (893, 970)	DUF1866 (EF03296)	Canonical RBD
Tap-RNA_bind (PF09162)	1FO1_A (123, 191)	Tap-RNA_bind (EF13157)	Non-canonical RBD domain*
MARF1_RRM1 (PF11608)	2DIU_A (8, 90)	Limkain-b1 (EF08767)	Limkain b1 domain-like
RRM_8 (PF11835)	2E5I_A (208, 293)	RRM_1_6 (EF24069)	No results
RRM_5 (PF13893)	2AD9_A (49, 146)	RRM_1_6 (EF24069)	No results
RRM_7 (PF16367)	2DNL_A (426, 515)	RRM_1_3 (EF21353)	Canonical RBD
RRM_occluded (PF16842)	2L9W_A (311, 393)	RRM_occluded (EF20754)	Occluded RRM-like*
YlmH_RBD (PF17774)	2FPH_X (82, 166)	YlmH_2nd (EF14865)	No results

Note:Representative StIs are randomly chosen from the list of 'True' domains for each of the listed Pfam family.
*No SCOP2 classification is available for given PDB ID explicitly.

2AD9_A (49, 146), 2FPH_X (82, 166)], do not have any classification in the SCOP database. The classification for other StIs in SCOP and all the StIs in ECOD, is in good agreement with CroMast results (Pfam and CATH).

F. RRM clan in Pfam

The RRM clan CL0221 from Pfam v35.0 contains families that are related to the RRM domain type and are thought to be evolutionarily related. This clan contains 33 families and the total number of domains in the clan is 433471. Table S3 lists all the families from RRM clan, their number of StIs (apart from obsolete or inconsistent ones) and their exploration by CroMaSt run1 (Main paper) and run2 (Section G, below).

G. CroMaSt run2 with 14 Pfam families from RRM clan

In a second run, we initiate the CroMaSt workflow with 14 Pfam families from RRM clan (13 Pfam families missing from the first run and PF00076) from Pfam and superfamily 3.30.70.330

Table S3. All the 33 Pfam families from RRM clan and their exploration by CroMaSt run1 (Main paper) and run2 (Supp. section G)

Family Ids	Family Name	No. of StIs	Explored by CroMaSt?	
			run1	run2
PF00076	RRM_1	1333	✓*	✓*
PF02994	Transposase_22	12	-	✓*
PF03467	Smg4_UPF3	3	✓	✓
PF03468	XS	2	-	✓*
PF03880	DbpA	4	✓	✓
PF04059	RRM_2	4	-	✓*
PF04847	Calcipressin	1	✓	✓
PF05172	Nup35_RRM	10	✓	✓
PF07576	BRAP2	No StI	-	-
PF08152	GUCT	2	-	✓*
PF08489	DUF1743	10	-	✓*
PF08675	RNA_bind	5	✓	✓
PF08777	RRM_3	4	✓	✓
PF08952	DUF1866	2	✓	✓
PF09162	Tap-RNA_bind	12	✓	✓
PF11608	MARF1_RRM1	1	✓	✓
PF11767	SET_assoc	No StI	-	-
PF11835	RRM_8	3	✓	✓
PF12220	U1snRNP70_N	20	-	✓*
PF13893	RRM_5	42	✓	✓
PF14605	Nup35_RRM_2	No StI	-	-
PF14703	PHM7_cyt	12	-	✓*
PF15023	DUF4523	No StI	-	-
PF15407	Spo7_2_N	No StI	-	-
PF16367	RRM_7	6	✓	✓
PF16842	RRM_occluded	6	✓	✓
PF17774	YlmH_RBD	1	✓	✓
PF17799	RRM_Rrp7	23	-	✓*
PF17797	RL	14	-	✓*
PF18440	GlcNAc-1_reg	1	-	✓*
PF18444	RRM_9	6	-	✓*
PF18528	Ret2_MD	3	-	✓*
PF19977	xRRM	2	-	✓*

*Starting Pfam family for CroMaSt workflow.

Table S4. Results from each step of CroMaSt workflow execution, starting with 14 Pfam families from RRM clan and CATH superfamily 3.30.70.330 (RRM domain)

Steps	Iteration 1		Iteration 2	
	Pfam	CATH	Pfam	CATH
Starting Families	14	1	14	3
StI filtered on domain length	1445	1527	117†	96†
Obsolete and inconsistent entries	1	323	0	0
Residue-mapped StIs	1444	1204	117	96
Common StIs (**'Core'** & **'True'**)	883	883	96	96
Remaining StIs (not common)	561	321	21	0
Cross-mapped StIs	21	243	0	0
Properly aligned at family level (**'True'**)	17	79	-	-
Not properly aligned at family level (**'Failed'**)	4*	164**	-	-
Un-mapped StIs	540	78	21	0
Properly aligned at instance level (**'Domain-like'**)	484	78	20	-
Not properly aligned at instance level (**'Failed'**)	56	0	1	-
Total **'Failed'** StIs	60	164	1	0
**'Failed'** domain families	3	1	0	0
New domain families for RRM domain type	14	3	0	0

*Corresponding to 1 CATH superfamily not assessed as RRM domain type.

**Corresponding to 3 Pfam families not assessed as RRM domain type.

†Some of these StIs are from last iteration (properly aligned at family level).

(RRM domain) from CATH. Table S4 shows the different results obtained at each step of the workflow.

A total of 1445 domain StIs were extracted from 14 Pfam families of which 1 was inconsistent, whereas 1527 domain StIs were extracted from CATH, of which 316 were obsolete and 7 inconsistent. Then, the 1444 and 1204 domain StIs from Pfam and CATH, respectively, were residue-mapped. Out of all these residue-mapped domain StIs, 883 are shared between Pfam and CATH. Thus, 883 StIs constitute the 'Core' domain StIs, and are also included in the list of 'True' domain StIs. Core average structure for RRM domain type was computed using these 883 StIs. These 883 'Core' domain StIs are the same as in the first run, resulting in the same core average structure. From the remaining StIs (561 unique to Pfam and 321 unique to CATH), 21 StIs from Pfam were successfully cross-mapped to 4 different CATH superfamilies and 243 StIs from CATH were successfully cross-mapped to a total of 17 different Pfam families. Thus, 'family-level' average structures were computed for these 4 and 17 newly found CATH and Pfam families using the cross-mapped StIs. After aligning these average structures against the core average structure, 17 (3 from CATH and 14 from Pfam) of them passed the threshold (M-score >= 0.6) allowing to include these families at the beginning of the next iteration. The remaining 4 families (1 from CATH and 3 from Pfam) and their corresponding StIs did not pass the structural alignment step (M-score < 0.6) and were considered as 'Failed' domain families and StIs. Thus, only 79 StIs from the 243 CATH StIs cross-mapped to Pfam and 14 new Pfam families were kept for the next iteration. Moreover, only 17 StIs from the 21 Pfam StIs cross-mapped to CATH and 3 new CATH superfamilies were kept for the next iteration.

The average structures were computed at the 'UniProt-instance-level' for all un-mapped StIs from Pfam (540) and CATH (78). After structural alignment against the core average structure, only 56 StIs from Pfam failed to pass the threshold. Thus, all remaining StIs (484 from Pfam, and 78 from CATH) are qualified as 'Domain-like' StIs.

In summary, the first iteration resulted in a total of 883 'Core', 562 'Domain-like', and 224 'Failed' domain StIs, as well as 3 CATH and 14 Pfam families along with 17 Pfam StIs and 79 CATH StIs ready for the next iteration.

The second iteration started with the 14 Pfam families (and 17 StIs from last iteration) and 3 CATH superfamilies (and 79 StIs from last iteration). A total of 100 (+17 from last iteration) domain StIs were extracted from the 14 Pfam families, whereas 17 (+ 79 from last iteration) domain StIs were extracted from 3 CATH superfamilies, with no inconsistent or obsolete entry.

The two sets shared 96 StIs ('True' domain StIs) and the other 21 StIs from Pfam remained un-mapped in CATH. Nearly all of them (20/21) passed the alignment threshold leading to 20 'Domain-like' StIs and 1 'Failed' domain StI. Thus, at the end of the second iteration, no new family was found, hindering any further iteration of the workflow.

In summary, the CroMaSt workflow, initialized with 14 Pfam families from RRM clan (including PF00076) and CATH superfamily 3.30.70.330, identified 979 'True' domain StIs (among which 883 are 'Core'), 582 'Domain-like', and 225 'Failed' domain StIs with respect to the RRM domain type. In terms of domain families, the CroMaSt workflow explored a total of 36 families (31 from Pfam and 5 from CATH, including the starting families) and 29 of them (25 from Pfam and 4 from CATH) qualified for the RRM domain type with at least one StIs in either 'True' or 'Domain-like' category (Table S4).

The 14 Pfam families explored by cross-mapping of CATH StIs in the first iteration of this run are same as in CroMaSt run1 (Main paper).

The 3 CATH superfamilies explored by cross-mapping of Pfam StIs in the first iteration of this run are 3.30.70.1820, 3.30.70.1970, 3.30.70.2280, and 3.90.600.20. The first three CATH superfamilies passed the alignment threshold and were included in the second iteration of the CroMaSt workflow, while the last superfamily (3.90.600.20) failed to pass the alignment threshold. The CATH superfamily 3.30.70.1820 (L1 transposable element, RRM domain) was the second superfamily found in CATH when searched with thr 'RRM' keyword (see Supp. section B).

Table S5. Results from each step of CroMaSt workflow execution, starting with Pfam family PF00028 (Cadherin) and CATH superfamily 2.60.40.60 (Cadherins)

Steps	Iteration 1		Iteration 2	
	Pfam	CATH	Pfam	CATH
Starting Families	1	1	3	0
StI filtered on domain length	785	454	92	40**
Obsolete and inconsistent entries	0	0	0	0
Residue-mapped StIs	785	454	92	–
Common StIs (**'Core'** & **'True'**)	397	397	40	40
Remaining StIs (not common)	388	57	52	0
Cross-mapped StIs	0	40	0	0
Properly aligned at family level (**'True'**)	–	40	–	–
Not properly aligned at family level (**'Failed'**)	–	0	–	–
Un-mapped StIs	388	17	52	0
Properly aligned at instance level (**'Domain-like'**)	388	17	52	–
Not properly aligned at instance level (**'Failed'**)	0	0	0	–
Total **'Failed'** StIs	0	0	0	0
**'Failed'** domain families	0	0	0	0
New domain families for RRM domain type	3	0	0	0

**Cross-mapped and properly aligned at the family level from the previous iteration.

H. CroMaSt runs for other domain types

H.1. Cadherin domain type

We instantiated the CroMaSt workflow with Pfam family PF00028 (Cadherin) and CATH superfamily 2.60.40.60 (Cadherins).

The results obtained at each step of the workflow are given in Table S5.

A total of 785 domain StIs were extracted from Cadherin Pfam family, whereas 454 domain StIs were extracted from CATH, with no inconsistent or obsolete entry. Then, all these domain StIs from Pfam and CATH, respectively, were residue-mapped. Out of all these residue-mapped domain StIs, 397 are shared between Pfam and CATH. Thus, 397 StIs constitute the 'Core' domain StIs, and are also included in the list of 'True' domain StIs. Core average structure for Cadherin domain type was computed using these 397 StIs. From the remaining StIs (388 unique to Pfam and 57 unique to CATH), only 40 StIs from CATH were successfully cross-mapped to a total of 3 different Pfam families. Thus, 'family-level' average structures were computed for these 3 Pfam families using the cross-mapped StIs. After aligning these average structures against the core average structure, all of them passed the threshold (M-score $>= 0.6$) allowing to include these families at the beginning of the next iteration. Thus, all domain StIs (40) cross-mapped to Pfam and 3 new Pfam families were kept for the next iteration.

The average structures were computed at the 'UniProt-instance-level' for all un-mapped StIs from Pfam (388) and CATH (17). After structural alignment against the core average structure, all StIs (388 from Pfam and 17 from CATH) are qualified as 'Domain-like' StIs.

In summary, the first iteration resulted in a total of 397 'Core', 405 'Domain-like', and 0 'Failed' domain StIs, with 3 Pfam families and 40 CATH StIs ready for the next iteration.

The second iteration started with the 3 Pfam families and 40 StIs from CATH. A total of 92 domain StIs were filtered from the 3 Pfam families, with no inconsistent or obsolete entry. The two sets shared 40 StIs ('True' domain StIs) and the other 52 StIs from Pfam remained un-mapped in CATH. All of them (52) passed the alignment threshold leading to 52 'Domain-like' StIs and no 'Failed' domain StI. Thus, at the end of the second iteration, no new family was found, hindering any further iteration of the workflow.

In summary, the CroMaSt workflow, initialized with Pfam PF00028 and CATH 2.60.40.60 domain families, identified 437 'True' domain StIs (among which 397 are 'Core'), 457 'Domain-like', and 0 'Failed' domain StIs with respect to the Cadherin domain type. In terms of domain families, the CroMaSt workflow explored a total of 5 families (4 from Pfam and 1 from CATH, including the starting families) and all of them (4 from Pfam and 1 from CATH) qualified for the Cadherin domain type (Table S5). The 3 Pfam families detected by cross-mapping of CATH StIs (PF08266, PF08758, and PF17756) are all three members of the same clan as of the starting family: CL0159 (E-set).

H.2. Zinc Finger domain type

We instantiated the CroMaSt workflow with Pfam family PF00096 (zf-C2H2) and CATH superfamily 3.30.160.60 (Classic Zinc Finger). We used the same parameters for this run of CroMaSt workflow except the starting family identifiers and

minimum domain length. We used a minimum domain length of 10 for the Zinc finger domain type because it is very small (∼25 aa) compared to Cadherin (∼93 aa) and RRM (∼90 aa) domain types. The results obtained at each step of the workflow are given in Table S6.

Table S6. Results from each step of CroMaSt workflow execution, starting with Pfam family PF00096 (zf-C2H2) and CATH superfamily 3.30.160.60 (Classic Zinc Finger)

Steps	Iteration 1		Iteration 2	
	Pfam	CATH	Pfam	CATH
Starting Families	1	1	4	0
StI filtered on domain length	579	359	45	7**
Obsolete and inconsistent entries	0	9	0	0
Residue-mapped StIs	579	350		-
Common StIs (**'Core'** & **'True'**)	217	217	7	7
Remaining StIs (not common)	362	133	38	0
Cross-mapped StIs	0	47	0	0
Properly aligned at family level (**'True'**)	-	7	-	-
Not properly aligned at family level (**'Failed'**)	-	40	-	-
Un-mapped StIs	362	86	38	0
Properly aligned at instance level (**'Domain-like'**)	362	58	38	-
Not properly aligned at instance level (**'Failed'**)	0	28	0	-
Total **'Failed'** StIs	0	68*	0	0
**'Failed'** domain families	10	0	0	0
New domain families for Zinc Finger domain type	4	0	0	0

*40 of these correspond to 10 Pfam domain families not assessed as Zinc Finger domain type.

**Cross-mapped and properly aligned at the family level from the previous iteration.

A total of 579 domain StIs were extracted from zf-C2H2 Pfam family, whereas 359 domain StIs were extracted from CATH, of which 8 were inconsistent and 1 obsolete. Then, the 579 and 350 domain StIs from Pfam and CATH, respectively, were residue-mapped. Out of all these residue-mapped domain StIs, 217 are shared between Pfam and CATH. Thus, 217 StIs constitute the 'Core' domain StIs, and are also included in the list of 'True' domain StIs. Core average structure for Zinc Finger domain type was computed using these 217 StIs. From the remaining StIs (362 unique to Pfam and 133 unique to CATH), only 47 StIs from CATH were successfully cross-mapped to a total of 14 different Pfam families. Thus, 'family-level' average structures were computed for these 14 Pfam families using the cross-mapped StIs. After aligning these average structures against the core average structure, 4 of them (corresponding to 7 cross-mapped StIs) passed the threshold (M-score >= 0.6) allowing to include these families at the beginning of the next iteration. The remaining 10 families and their corresponding StIs (40) did not pass the structural alignment step (M-score < 0.6) and were considered as 'Failed' domain families and StIs. Thus, only 7 domain StIs from 47 CATH StIs cross-mapped to Pfam were kept for the next iteration.

The average structures were computed at the 'UniProt-instance-level' for all un-mapped StIs from Pfam (362) and CATH (86). After structural alignment against the core average structure, only 28 StIs from CATH failed to pass the threshold. Thus all remaining StIs (362 from Pfam and 58 from CATH) are qualified as 'Domain-like' StIs.

In summary, the first iteration resulted in a total of 217 'Core', 420 'Domain-like', and 68 'Failed' domain StIs, with 4 Pfam families and 7 CATH StIs ready for the next iteration.

The second iteration started with the 4 Pfam families and 7 StIs from CATH. A total of 45 domain StIs were filtered from the 4 Pfam families, with no inconsistent or obsolete entry. The two sets shared 7 StIs ('True' domain StIs) and the other 38 StIs from Pfam remained un-mapped in CATH. All of them (38) passed the alignment threshold leading to 38 'Domain-like' StIs and no 'Failed' domain StI. Thus, at the end of the second iteration, no new family was found, hindering any further iteration of the workflow.

In summary, the CroMaSt workflow, initialized with Pfam PF00096 and CATH 3.30.160.60 domain families, identified 224 'True' domain StIs (among which 217 are 'Core'), 458 'Domain-like', and 68 'Failed' domain StIs with respect to the Zinc Finger domain type. In terms of domain families, the CroMaSt workflow explored a total of 16 families (15 from Pfam and 1 from CATH, including the starting families) and 6 of them (5 from Pfam and 1 from CATH) qualified for the Zinc Finger domain type (Table S6).

The 4 Pfam families detected by cross-mapping of CATH StIs (PF08209, PF10426, PF13909, PF18450) are all three members of the same clan as of the starting family: CL0361 (C2H2-zf). However, some of the 10 'Failed' Pfam families are also from the same clan. This suggests that the alignment threshold used is too high and should be lowered in order to retrieve more 'True' domain StIs from different families assigned to this domain type. It is likely because of the rather small size (about 25 amino acids) of this domain type.

References

A. Andreeva, E. Kulesha, J. Gough, and A. G. Murzin. The SCOP database in 2020: expanded classification of representative family and superfamily domains of known protein structures. *Nucleic Acids Res*, 48(D1):D376–D382, 01 2020.

M. Blum, H. Y. Chang, S. Chuguransky, T. Grego, S. Kandasaamy, A. Mitchell, G. Nuka, T. Paysan-Lafosse, M. Qureshi, S. Raj, L. Richardson, G. A. Salazar, L. Williams, P. Bork, A. Bridge, J. Gough, D. H. Haft, I. Letunic, A. Marchler-Bauer, H. Mi, D. A. Natale, M. Necci, C. A. Orengo, A. P. Pandurangan, C. Rivoire, C. J. A. Sigrist, I. Sillitoe, N. Thanki, P. D. Thomas, S. C. E. Tosatto, C. H. Wu, A. Bateman, and R. D. Finn. The InterPro protein families and domains database: 20 years on. *Nucleic Acids Res*, 49(D1):D344–D354, 01 2021.

H. Cheng, R. D. Schaeffer, Y. Liao, L. N. Kinch, J. Pei, S. Shi, B.-H. Kim, and N. V. Grishin. Ecod: an evolutionary classification of protein domains. *PLoS computational biology*, 10(12):e1003926, 2014.

S. Lu, J. Wang, F. Chitsaz, M. K. Derbyshire, R. C. Geer, N. R. Gonzales, M. Gwadz, D. I. Hurwitz, G. H. Marchler, J. S. Song, N. Thanki, R. A. Yamashita, M. Yang,

D. Zhang, C. Zheng, C. J. Lanczycki, and A. Marchler-Bauer. CDD/SPARCLE: the conserved domain database in 2020. *Nucleic Acids Res*, 48(D1):D265–D268, 01 2020.

J. Mistry, S. Chuguransky, L. Williams, M. Qureshi, G. A. Salazar, E. L. L. Sonnhammer, S. C. E. Tosatto, L. Paladin, S. Raj, L. J. Richardson, R. D. Finn, and A. Bateman. Pfam: The protein families database in 2021. *Nucleic Acids Res*, 49(D1):D412–D419, 01 2021.

T. Paysan-Lafosse. Pfam website decommission. `https://xfam.wordpress.com/2022/08/04/pfam-website-decommission/`, 2022. Accessed: 2022-09-03.

I. Sillitoe, N. Bordin, N. Dawson, V. P. Waman, P. Ashford, H. M. Scholes, C. S. M. Pang, L. Woodridge, C. Rauer, N. Sen, M. Abbasian, S. Le Cornu, S. D. Lam, K. Berka, I. H. Varekova, R. Svobodova, J. Lees, and C. A. Orengo. CATH: increased structural coverage of functional space. *Nucleic Acids Res*, 49(D1):D266–D273, 01 2021.
